# The CRH‐Transgenic Cushingoid Mouse Is a Model of Glucocorticoid‐Induced Osteoporosis

**DOI:** 10.1002/jbm4.10009

**Published:** 2017-06-28

**Authors:** Jasmine Williams‐Dautovich, Keertika Yogendirarajah, Ariana Dela Cruz, Rucha Patel, Ricky Tsai, Stuart A Morgan, Jane Mitchell, Marc D Grynpas, Carolyn L Cummins

**Affiliations:** ^1^ Department of Pharmaceutical Sciences University of Toronto Toronto Ontario Canada; ^2^ Lunenfeld‐Tanenbaum Research Institute Mount Sinai Hospital Toronto Ontario Canada; ^3^ Department of Pharmacology and Toxicology University of Toronto Toronto Ontario Canada; ^4^ Department of Laboratory Medicine and Pathobiology University of Toronto Toronto Ontario Canada

**Keywords:** CUSHING'S DISEASE, GENETIC ANIMAL MODELS, CORTICOSTEROIDS, BONE HISTOMORPHOMETRY, μCT

## Abstract

Glucocorticoids (GCs) have unparalleled anti‐inflammatory and immunosuppressive properties, which accounts for their widespread prescription and use. Unfortunately, a limitation to GC therapy is a wide range of negative side effects including Cushing's syndrome, a disease characterized by metabolic abnormalities including muscle wasting and osteoporosis. GC‐induced osteoporosis occurs in 30% to 50% of patients on GC therapy and thus, represents an important area of study. Herein, we characterize the molecular and physiologic effects of GC‐induced osteoporosis using the Cushing's mouse model, the corticotropin releasing hormone (CRH) transgenic mouse (CRH‐Tg). The humeri, femurs, and tibias from wild‐type (WT) and CRH‐Tg male mice, aged 13 to 14 weeks old were subjected to multiple bone tests including, micro–computed tomography (μCT), static and dynamic histomorphometry, strength testing, and gene expression analyses. The CRH‐Tg mice had a 38% decrease in cortical bone area, a 35% decrease in cortical thickness, a 16% decrease in trabecular thickness, a sixfold increase in bone adiposity, a 27% reduction in osteoid width, a 75% increase in bone‐resorbing osteoclast number/bone surface, a 34% decrease in bone formation rate, and a 40% decrease in bone strength compared to WT mice. At the gene expression level, CRH‐Tg bone showed significantly increased osteoclast markers and decreased osteoblast markers, whereas CRH‐Tg muscle had increased muscle atrophy gene markers compared to WT mice. Overall, the CRH‐Tg mouse model aged to 14 weeks recapitulated many features of osteoporosis in Cushing's syndrome and thus, represents a useful model to study GC‐induced osteoporosis and interventions that target the effects of GCs on the skeleton. © 2017 The Authors. *JBMR Plus* is published by Wiley Periodicals, Inc. on behalf of the American Society for Bone and Mineral Research.

## Introduction

It is estimated that between 0.5% to 1.2% of the global population is currently being treated with glucocorticoid (GC) drugs.^1^ GC drugs are first‐line therapy for many inflammatory conditions such as inflammatory bowel disease, asthma, rheumatoid arthritis, and chronic obstructive pulmonary disease. Unfortunately, an ongoing limitation to therapeutic GC use is the myriad of negative side effects including the development of Cushing's syndrome.^2^, ^3^ Cushing's syndrome can result from exogenous administration of GC drugs or it can arise from endogenous over production of cortisol, usually secondary to an adrenocorticotropic hormone (ACTH)‐secreting pituitary tumor. Features of Cushing's syndrome include central obesity, hyperglycemia, osteoporosis, and muscle wasting.^4^, ^5^

GC‐induced osteoporosis (GIO) is characterized by an initial stage of rapid bone loss, followed by a slower, but consistent decrease in bone mass. GIO results in bone fractures in 30% to 50% of patients, which can lead to increased morbidity, mortality, and reduced quality of life.^6^, ^7^ GIO is characterized by low bone density and microarchitectural changes that occur as a result of GCs acting on the three main bone cell types: osteoblasts, osteoclasts, and osteocytes. GCs inhibit osteoblast differentiation and promote osteoblast apoptosis. This results in reduced bone formation, which is the primary factor leading to GIO.^8^ GCs also cause apoptosis of osteocytes, the mechanosensing cells embedded throughout the bone matrix, resulting in decreased bone strength.^9^ Bone resorbing osteoclasts increase in number in response to GCs due to increased differentiation and decreased apoptosis, and this leads to increased bone turnover.^8^ In addition to the direct effects of GCs on bone cells, GCs also inhibit intestinal calcium absorption, leading to further pressure on bone calcium stores, resulting in decreased bone mass. Finally, GCs cause muscle atrophy, leading to decreased loading of bone and is associated with thinner bones.^10^ This combination of increased bone resorption and decreased bone formation results in an overall reduction in bone density, bone architecture, and bone cellularity, which all contribute to fracture risk.^11^ Trabecular bone is thought to be more sensitive to the anti‐osteogenic effects of GCs compared to cortical bone based on changes in bone mineral density. In line with this, there is a higher incidence of fractures in the femoral neck and vertebrae; compartments that have a higher percentage of trabecular bone.^6^ However, cortical bone is also significantly affected by GCs,^12^, ^13^ and a recent study of Cushing's syndrome patients using micro–computed tomography (μCT) for analysis revealed a dominant effect of GCs on cortical bone microstructure.^14^ It is therefore important to consider all parameters in parallel when studying the effects of GCs on the skeleton.

The mouse model of Cushing's syndrome used in this study was developed in 1992 and is transgenic for the rat corticotropin releasing hormone (CRH) gene downstream of the metallothionein promoter.^15^ Overexpression of CRH induces the release of ACTH from the pituitary gland, which then stimulates the production of corticosterone from the adrenal gland. This results in overstimulation of the hypothalamic‐pituitary‐adrenal (HPA) axis and thus chronic corticosterone production. The CRH‐Tg mice are on a C57BL/6J background and were reported to have fivefold increased plasma ACTH and 10‐fold increased plasma corticosterone levels compared to wild‐type (WT) mice.^15^ A previous study investigated the phenotype of these mice and found that CRH‐Tg mice have reduced whole‐body bone mineral density (BMD) as well as reduced bone volume and cortical thickness, as measured by μCT in the femur.^16^ Herein, we report bone histomorphometry, bone mechanical strength testing, and molecular analysis by gene expression of bone and muscle in the CRH‐Tg versus WT mice. This extended phenotyping provides a baseline from which studies investigating therapies for the treatment of GIO resulting from Cushing's syndrome can be performed.

## Materials and Methods

### Animal experiments

CRH‐Tg mice were obtained by reconstitution of cryopreserved embryos from the Jackson Laboratory (Bar Harbor, ME, USA; C57BL/6‐Tg(Crh)227.1Pbl/J; Stock #003210). The cryo‐recovered CRH‐Tg (hemizygous) males were bred two to three generations with C57Bl/6 females from our colony to generate sufficient CRH males, and corresponding WT littermate controls for the experiment. Verification of the C57Bl/6 background of the CRH litters was carried out by the Jackson Laboratory Genome Scanning Service. Results showed that the generated CRH litters and C57Bl/6 female breeders showed a 99% similarity in background. Litters were genotyped following the Jackson Laboratory protocol for the CRH transgene.

Mice were housed in a pathogen‐free, temperature‐controlled facility with a 10‐hour/14‐hour light/dark cycle. All mice were fed the Teklad 2016 global diet (Envigo, Mississauga, Canada) *ad libitum*. Male, 13‐week‐old to 14‐week old, CRH‐Tg (*n* = 6) and WT (*n* = 9) littermate control mice were euthanized by decapitation within 6 hours after the start of the light cycle. The gastrocnemius muscle and humeri bone samples were flash frozen in liquid nitrogen. Trunk blood was collected in tubes containing 5 μL of 0.5M EDTA and plasma was separated by centrifugation. Dynamic histomorphometry was measured in a separate cohort of male 14‐week‐old mice that had been injected with 30 mg/kg calcein green (Sigma‐Aldrich, Oakville, Canada) 7 and 2 days before euthanasia. These mice served as control groups for a separate experiment and were all injected s.c. with 30 μL of 5% ethanol in sesame oil twice daily for 6 weeks. All animal experiments performed were approved by the Faculty of Medicine and Pharmacy Animal Care Committee at the University of Toronto in accordance with the guidelines from the Canadian Council on Animal Care. Although the genotype of the mice was apparent at euthanasia (because of the thinning of the fur for the CRH‐Tg mice), once the bones were removed and cleaned for further processing, all samples were only identified by number. The μCT, three‐point bending and histomorphometry analyses were performed in a blinded manner.

### RNA analysis, cDNA synthesis, and real‐time quantitative PCR analysis

For RNA analysis, humeri were flash frozen in liquid nitrogen without removal of the bone marrow. Tissues were homogenized in RNA STAT‐60 (Tel‐Test, Friendswood, TX, USA) to extract total RNA and reverse transcribed into cDNA with random hexamer primers using the High Capacity Reverse Transcription System (Applied Biosystems, Carlsbad, CA, USA). Real‐time quantitative PCR (qPCR) reactions were performed in 384‐well plates containing 12.5 ng cDNA, 150 nM of each primer and 5 μL 2× SYBR Green Master Mix in a 10‐μL total volume. The comparative threshold cycle (Ct) method was used to determine relative mRNA levels normalized to Cyclophilin or Gapdh mRNA, as indicated. The qPCR primers used are listed in Table 1.

**Table 1:** qPCR Primer Sequences

Mouse gene	Accession#	Forward and reverse primers (5′–3′)
*Acp5*	NM_007388.3	CTAAGATCTCCAAGCGCTGGAA GGAATTTTGAAGCGCAAACG
*Bglap*	NM_001037939.1	GGCCCTGAGTCTGACAAAGC GCCGGAGTCTGTTCACTACCTT
*Col1a1*	NM_007742.3	ACGTCTGGTTTGGAGAGAGCA TGAGCCCTCGCTTCCGTACT
*Ctsk*	NM_007802.4	GTTGTATGTATAACGCCACGGC CTTTCTCGTTCCCCACAGGA
*Cyclophilin*	NM_000942.4	GGAGATGGCACAGGAGGAA GCCCGTAGTGCTTCAGTTT
*Fbxo32*	NM_026346.3	CTTCCATCAAGAAAAGTGGATCTATG AAAGCTTCCCCCAAAGTACAGTATC
*Foxo1*	NM_019739.3	TCATGGATGGAGATACCTTGGA CTTGACACTGTGTGGGAAGCTT
*Gapdh*	NM_008084.2	GACGGCCGCATCTTCTTGT ACCGACCTTCACCATTTTGTCT
*Mstn*	NM_010834.2	CTACCACGGAAACAATCATTACCAT TGCCATCCGCTTGCATT
*Opg*	NM_008764.3	CCACGCAAAAGTGTGGAATAGA GGTAGGAACAGCAAACCTGAAGA
*Oscar*	NM_001290377.1	ACCTGGCACCTACTGTTGCTATTACG CTGCGCTGTGATAGCACAT
*Ppparg2*	NM_001127330.2	TCGCTGATGCACTGCCTATG GAGAGGTCCACAGAGCTGATT
*Rankl*	NM_011613.3	GCACACCTCACCATCAATGC TGGTACCAAGAGGACAGAGTGACTT
*Runx2*	NM_001145920.2	TCTGGCCTTCCTCTCTCAGTAAGA GGCTCTCAGTGAGGGATGAAAT
*Trim63*	NM_001039048.2	AACACAACCTCTGCCGGAAGT CCGCGGTTGGTCCAGTAG

### Histology

The right tibias were fixed immediately after euthanasia in 70% ethanol at 4°C, followed by acetone dehydration, infiltration with ascending concentrations of Spurr resin, and embedding in Spurr resin. A Leica RM2265 microtome (Leica Microsystems, Richmond Hill, Canada) was used to cut 5‐μm‐thick sections with a tungsten carbide knife. The undecalcified 5‐μm sections of the right tibia were stained with Goldner's Trichrome stain and imaged with magnification ×200 for analysis.

The left tibias were fixed in 10% neutral buffered formalin. The bones were then decalcified in 0.5M EDTA, pH 7.4, at 4°C. After decalcification, the specimens were processed in a graded series of ethanol and xylene, and embedded in paraffin wax. Five‐μm‐thick (5‐μm‐thick) sections were cut using a Reichart‐Jung 2030 microtome (Boston, MA, USA). The decalcified sections were incubated in 0.2M Tris buffer (Trizma base, pH 9; Sigma‐Aldrich) for 1 hour and then stained for tartrate‐resistant acid phosphatase (TRAP) using the Acid Phosphatase Leukocyte kit (Sigma‐Aldrich), following the manufacturer's instructions. The TRAP slides were imaged under magnification ×100 for analysis. Undecalcified 7‐μm‐thick sections were used for dynamic calcein histomorphometry to measure bone formation.

Histomorphometric data was quantified using Bioquant Osteo v13.2 MIR software (Bioquant Image Analysis Corporation, Nashville, TN, USA). The regions of interest (ROIs) for analysis of trabecular bone were measured 150 μm under the growth plate in the secondary spongiosa of the proximal tibial metaphysis. In undecalcified sections, the parameters measured were the following: percent bone volume (BV/TV), trabecular thickness (Tb.Th), trabecular number (Tb.N), trabecular separation (Tb.Sp), osteoid surface per bone surface (OS/BS), osteoid volume per bone volume (OV/BV), osteoid width (O.Wi), number of osteoblasts per bone surface (N.Ob/BS), adipocyte volume per tissue volume (Ad.V/TV), mineralizing surface per bone surface (MS/BS), bone formation rate per bone surface (BFR/BS), and mineral apposition rate (MAR). In decalcified sections, the following were measured: number of osteoclasts per bone surface (N.Oc/BS) and number of osteoclasts per osteoclast surface (N.Oc/Oc.S). All histomorphometric measurements were expressed following the guidelines of the American Society of Bone and Mineral Research (ASBMR).^17^

### BMD and bone architecture

Left femurs were scanned in saline‐soaked gauze using a Skyscan 1174 compact desktop micro‐computed tomography (Bruker μCT; Skyscan, Kontich, Belgium) to measure the volumetric bone mineral density (vBMD) and microarchitecture of the femurs. A set of two hydroxyapatite phantoms were also scanned for BMD calibration. For trabecular bone assessment, the distal femur metaphysis was scanned at a voxel size of 6.1 μm and for cortical bone assessment, the femur mid‐diaphysis was scanned at a voxel size of 11.6 μm. The acquisition of images was performed at a 50 kV voltage, with 800 μA and a 0.25‐mm aluminum filter to reduce image noise. Image reconstruction was performed using the Skyscan NRecon software (version 1.6.3.2) and then analyzed by the Skyscan CT analyzer (version 1.10.0.1). For trabecular bone analysis, a volume of interest (VOI) was calculated 200 sections proximally from the formation of the first cartilage bridge, with a VOI of 100 sections (0.6 mm). Cortical bone analysis was performed on the femur mid‐diaphysis in order to obtain an 86‐section‐thick (1 mm) VOI. The parameters measured by μCT were as follows: BV/TV, Tb.Th, Tb.N, Tb.Sp, trabecular vBMD, mean total cross‐sectional bone area (B.Ar), cross‐sectional thickness (Cs.Th), cortical volumetric bone mineral density (Cortical vBMD), mean mediolateral diameter (ML), mean anteroposterior diameter (AP), and mean cortical thickness (Ct.Th).

### Three‐point bending

The left femurs were subjected to a three‐point bending test with the use of the Instron 4465 machine (Instron, Norwood, MA, USA). Initially, the femur lengths were measured with digital calipers to acquire their mid‐point, and other geometrical properties were obtained from the μCT imaging. The specimens were thawed to room temperature, rehydrated with saline, and then placed on two supports at a distance of 6 mm. A load of 100 N was applied to the mid‐point of the femurs at a rate of 0.5 mm/min until failure. The load‐deformation data was recorded using the LabVIEW 5.0 Acquisition Software (National Instruments, Austin, TX, USA) and normalized parameters were computed using the necessary femoral geometry data acquired from μCT analyses. The resulting structural parameters are ultimate load, energy to fail, and stiffness, and the material parameters are ultimate stress, toughness, and modulus.

### Plasma analysis

ELISA kits were used to measure osteocalcin (#60‐1305; Immunotopics, San Clemente, CA, USA) and TRAP‐5b (SB‐TR103; Immunodiagnostics, Gaithersburg, MD, USA) from plasma. Corticosterone was measured by radioimmunoassay (#07120002; MP Biomedical, Santa Ana, CA, USA).

### Statistical analyses

Data are presented as mean ± SE. GraphPad PRISM was used to perform the *t* test with Welch's correction (GraphPad Software, Inc., La Jolla, CA, USA). Outliers were detected by the ROUT method and removed. Outliers were detected in Fig.  2 and Table 2, as described in their respective legends; *p* < 0.05 was considered statistically significant.

**Table 2:** Mechanical Testing by 3‐Point Bending of Femurs in WT and CRH‐Tg Mice

	WT	CRH‐Tg	*p*
Structural properties			
Ultimate load (N)	16 ± 1	9.8 ± 0.4^b^, *	0.0019
Energy to fail (mJ)	16 ± 2	9.4 ± 0.5^b^, *	0.0156
Stiffness (N/mm)	44 ± 4	27 ± 3^b^, *	0.0069
Material properties			
Ultimate stress (MPa)	153 ± 8	136 ± 10	0.2280
Toughness (MPa)	32 ± 4^a^	27 ± 2	0.2722
Modulus (MPa)	1820 ± 142	1856 ± 202	0.8905

Data represent the average ± SE, *n* = 9 for WT and *n* = 6 for CRH‐Tg.

^a^ One outlier was removed (*n* = 8).

^b^ One outlier removed (*n* = 5).

* *p* < 0.05 WT versus CRH‐Tg using *t* test with Welch's correction.

## Results

### CRH‐Tg mice have similar body weights but increased white adipose tissue compared to WT littermate controls

**Figure 1:**
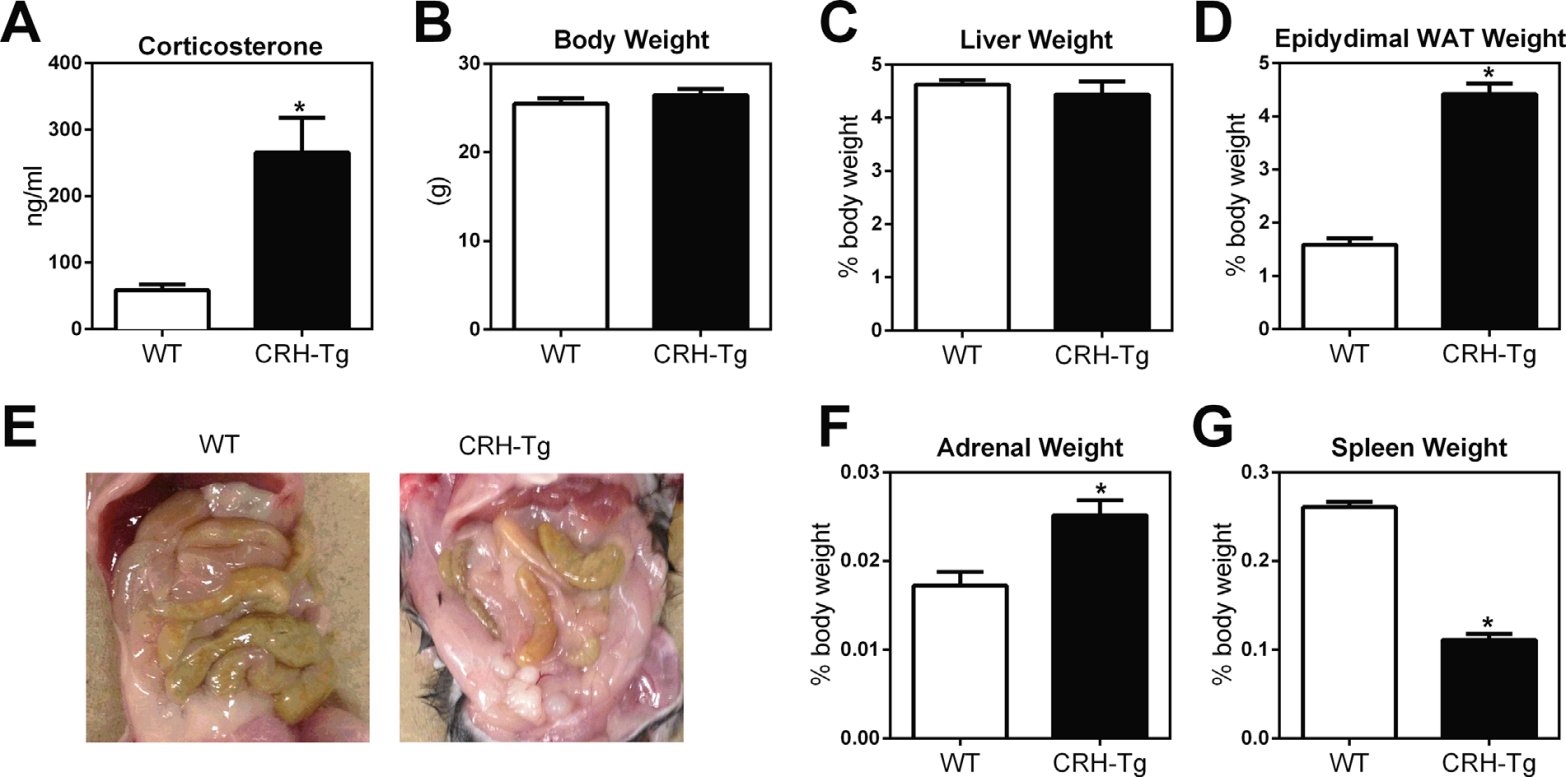
CRH‐Tg mice display features of Cushing's syndrome such as increased adiposity and lymphoid organ atrophy. WT and CRH‐Tg mice at 13‐14 weeks of age were compared for differences in (*A*) plasma corticosterone, (*B*) body weight, (*C*) liver weight, (*D*) epididymal WAT weight, (*E*) overall adiposity in the abdominal cavity, (*F*) adrenal weight, and (*G*) spleen weight. Data represent the average ± SE; WT: *n* = 9 and CRH‐Tg: *n* = 6. *p < 0.05 WT versus CRH‐Tg using *t* test with Welch's correction. WAT = white adipose tissue.

At 13 to 14 weeks of age, male CRH‐Tg mice had fivefold increased plasma corticosterone levels compared to WT mice (Fig. 1 *A*). There was no difference between genotypes in total body weight (Fig. 1 *B*) and liver weight (Fig. 1 *C*). In contrast, epididymal white adipose tissue (WAT) weights and overall abdominal adiposity were significantly increased in CRH‐Tg mice compared to WT mice (Fig. 1 *D*, *E*). Adrenal weights were also significantly increased in CRH‐Tg mice, likely due to chronic ACTH stimulation in response to CRH overexpression (Fig. 1 *F*). Spleen weights were decreased in CRH‐Tg mice, as expected, because corticosterone is known to promote lymphoid organ atrophy (Fig. 1 *G*).

### Cortical morphometry parameters and BMD are decreased in CRH‐Tg mice

**Figure 2:**
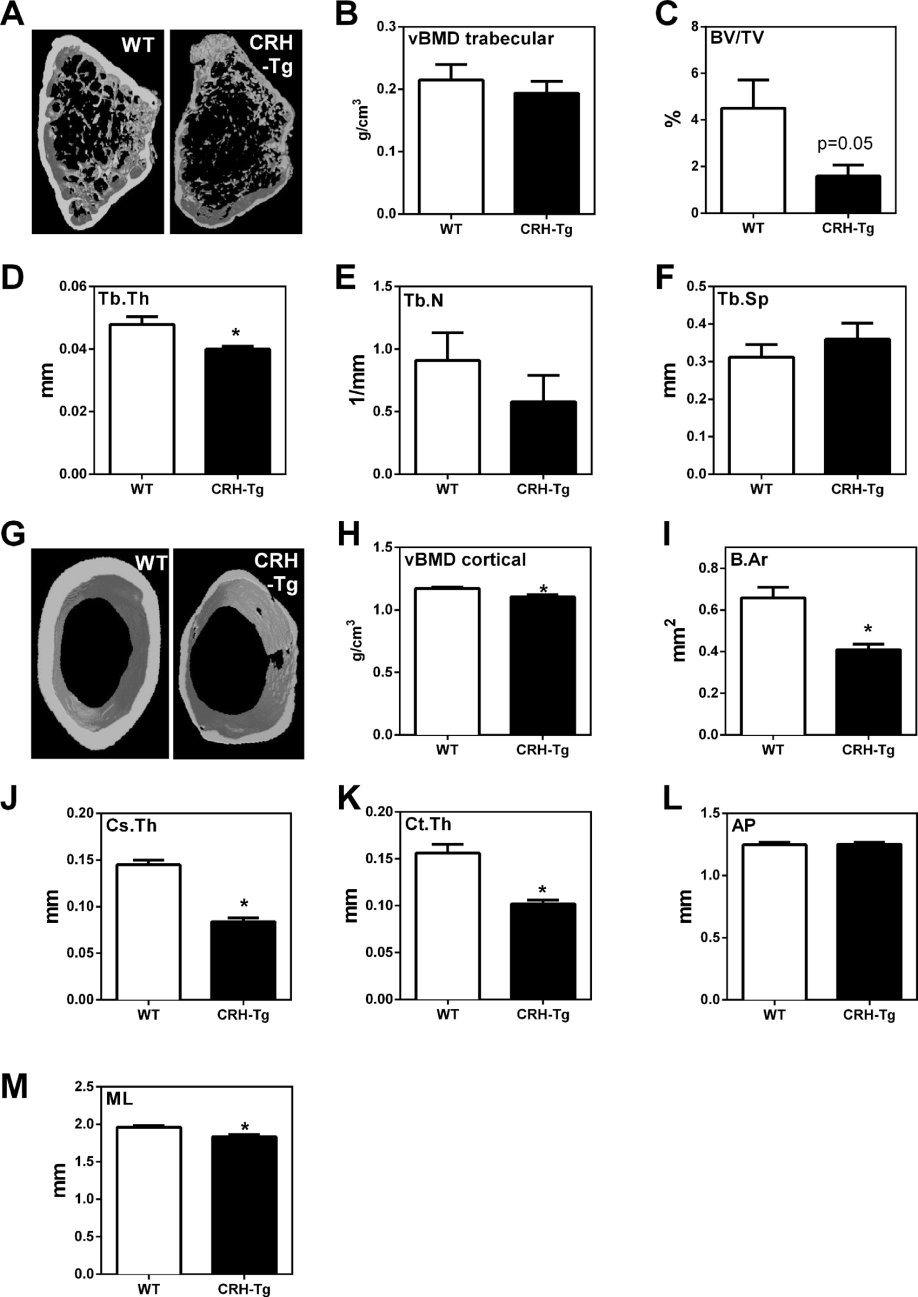
Cortical and trabecular bone parameters are altered in CRH‐Tg mice relative to WT mice and CRH‐Tg mice at 13 to 14 weeks of age were compared for differences using μCT of trabecular (*A*–*F*) and cortical (*G*–*M*) bone compartments in the femur. (*A*) Representative image of trabecular bone reconstruction, (*B*) vBMD of trabecular bone, (*C*) BV/TV, (*D*) Tb.Th, (*E*) Tb.N, and (*F*) Tb.Sp. (*G*) Representative image of cortical bone reconstruction, (*H*) vBMD of cortical bone, (*I*) B.Ar, (*J*) Cs.Th, (*K*) Ct.Th, (*L*) AP, and (*M*) ML. Data were obtained from 13‐week‐old to 14‐week‐old male WT (*n* = 8 for trabecular and *n* = 9 for cortical parameters) and CRH‐Tg (*n* = 6) mice and represent the average ± SE. *p < 0.05 WT versus CRH‐Tg using *t* test with Welch's correction. One WT femur was broken around the area of interest for trabecular analysis and could not be analyzed. One outlier was removed from the WT group for *I*, *J*, *M*. One outlier was removed from CRH‐Tg group for *C*, *D*, *I*, *J*, *K*. vBMD = volumetric BMD; BV/TV = % bone volume; Tb.Th = trabecular thickness; Tb.N = trabecular number; Tb.Sp = trabecular separation; B.Ar = bone area; Cs.Th = cross‐sectional thickness; Ct.Th = cortical thickness; AP = anteroposterior diameter; ML = mediolateral diameter.

Cortical and trabecular parameters of the femur were measured by μCT. Representative images of trabecular bone from WT and CRH‐Tg mice are shown in Fig. 2 *A*. The trabecular vBMD g/cm^3^ of CRH‐Tg mice compared to WT mice was not significantly altered (*p* = 0.5; Fig. 2 *B*). However, there was a trend toward a decrease in percent bone volume (*p* = 0.05; Fig. 2 *C*) and a 16% decrease in trabecular thickness (*p* = 0.02; Fig. 2 *D*) in CRH‐Tg mice compared to WT. In contrast, trabecular number was unchanged between CRH‐Tg and WT mice (*p* = 0.3; Fig. 2 *E*). Trabecular separation, a measure of the distance between the trabeculae, was also unchanged between genotypes (*p* = 0.4; Fig. 2 *F*). Representative images of cortical bone from WT and CRH‐Tg femurs are shown in Fig. 2 *G*. The vBMD of the cortical bone from CRH‐Tg mice was significantly lower than that of WT mice (*p* = 0.01; Fig. 2 *H*). Consistent with this, cortical bone from CRH‐Tg mice had a 38% reduction in bone area (*p* = 0.001; Fig. 2 *I*), a 42% reduction in cross‐sectional thickness (*p* < 0.001; Fig. 2 *J*), and a 35% reduction in cortical thickness compared to WT mice (*p* < 0.001; Fig. 2 *K*). Anteroposterior diameter was unchanged (*p* = 0.8; Fig. 2 *L*), but mediolateral diameter was decreased in CRH‐Tg mice (*p* = 0.007; Fig. 2 *M*), suggesting minor changes to the cortical shape by chronic GC exposure. Together, these data show that although parameters in both the trabecular and cortical compartments are significantly impacted by chronic GCs, the cortical compartment is more significantly affected by chronic GC exposure in this model.

### CRH‐Tg mice have increased bone adiposity, reduced osteoid, increased osteoclast numbers, and decreased bone formation rates compared to WT mice

**Figure 3:**
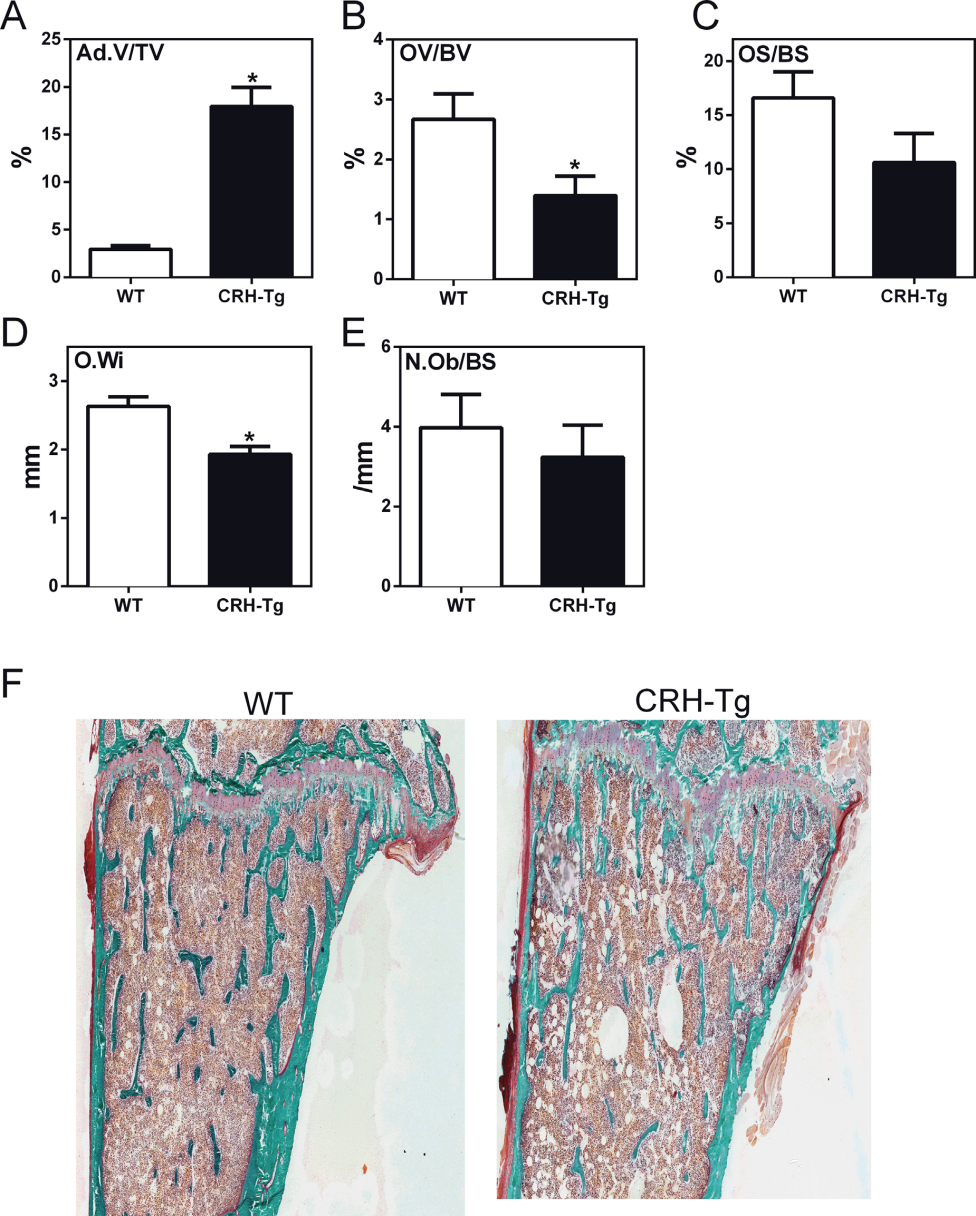
Adipocytes are significantly increased in the bone of CRH‐Tg mice compared to WT mice. Static histomorphometry of the proximal tibial metaphysis with trichrome staining of 13‐week‐old to 14‐week‐old male WT (*n* = 9) and CRH‐Tg (*n* = 6) mice. Measurements are (*A*) Ad.V/TV, (*B*) OV/BV, (*C*) OS/BS, (*D*) O.Wi, and (*E*) N.Ob/BS. (*F*) Representative image of trichrome staining of tibia. Data represent the average ± SE. *p < 0.05 WT versus CRH‐Tg using *t* test with Welch's correction. Ad.V/TV = adipocyte volume per tissue volume; OV/BV = percent osteoid volume per bone volume; OS/BS = percent of bone surface covered with osteoid; O.Wi = osteoid width; N.Ob/BS = number of osteoblasts per bone surface.

**Figure 4:**
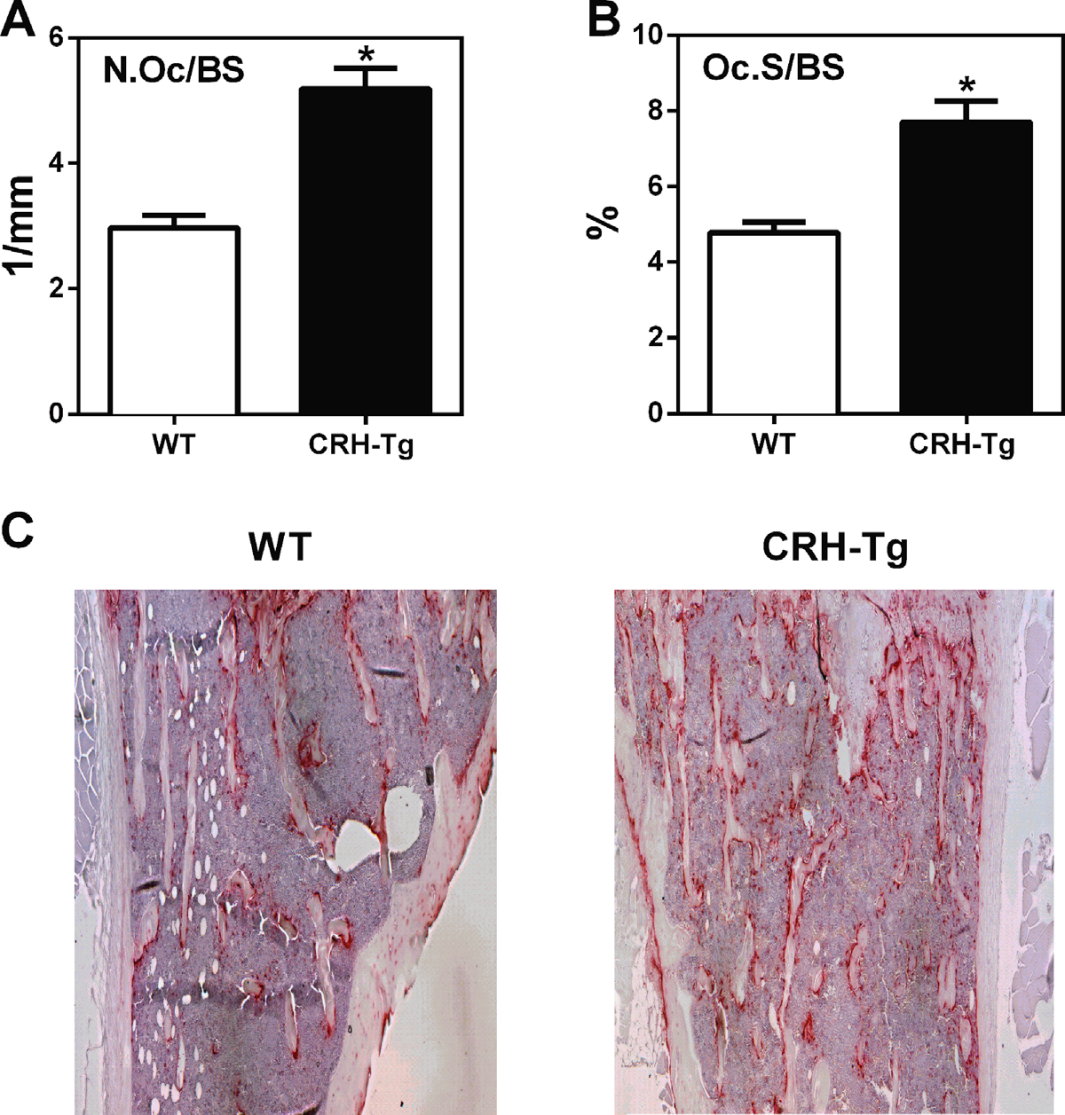
CRH‐Tg mice have an increase in osteoclasts compared to WT mice. Static histomorphometry of the proximal tibial metaphysis of 13‐week‐old to 14‐week‐old male WT (*n* = 9) and CRH‐Tg (*n* = 6) mice stained with the osteoclast marker TRAP. Measurements are (*A*) N.Oc/BS and (*B*) Oc.S/BS. Representative image from WT and CRH‐Tg mice tibias stained with TRAP (*C*). Data represent the average ± SE. *p < 0.05 WT versus CRH‐Tg using *t* test with Welch's correction. N.Oc/BS = number of osteoclasts per bone surface; Oc.S/BS = osteoclast surface per bone surface.

In order to elucidate the cellular mechanisms behind the changes in bone parameters, static histomorphometry of proximal tibial metaphyses was performed. Remarkably, adipocyte volume was increased sixfold in the bones of CRH‐Tg mice when compared to WT mice (*p* = 0.0006; Fig. 3 *A*). Osteoid volume to bone volume was decreased 47% in CRH‐Tg mice compared to WT (*p* = 0.03; Fig. 3 *B*). Osteoid surface to bone surface showed a trend toward a reduction in CRH‐Tg mice (*p* = 0.12; Fig. 3 *C*), whereas osteoid width was decreased by 27% in CRH‐Tg mice relative to WT mice (*p* = 0.002; Fig. 3 *D*). In contrast, the number of osteoblasts per bone surface was not different between the two genotypes (*p* = 0.5; Fig. 3 *E*). Representative trichrome stained tibial sections are shown in Fig. 3 *F*. Next, we stained for osteoclasts (OCs) using the OC marker TRAP. TRAP staining revealed a 75% increase in OC number per bone surface (*p* = 0.0003; Fig. 4 *A*) and a 61% increase in OC surface per bone surface in CRH‐Tg mice compared to WT mice (*p* = 0.0018; Fig. 4 *B*). A representative example of TRAP staining in WT and CRH‐Tg is shown in Fig. 4 *C*. Together, these results show that the CRH‐Tg mice have increased bone adiposity and increased osteoclasts, with an overall decrease in osteoblast‐associated osteoid width and volume, reflecting a decrease in bone formation.

**Figure 5:**
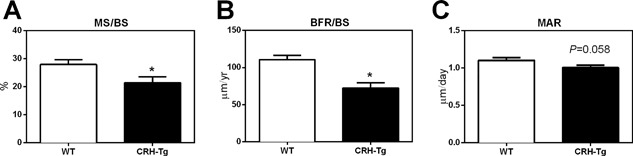
CRH‐Tg mice have reduced bone formation compared to WT mice. Dynamic histomorphometric analysis of proximal tibia from 14‐week old WT and CRH‐Tg mice after dual calcein labeling measuring (*A*) MS/BS; (*B*) BFR/BS; and (*C*) MAR. Data represent average ± SE, *n* = 10 to 11. *p < 0.05 WT versus CRH‐Tg using *t* test with Welch's correction. MS/BS = mineralizing surface per bone surface; BFR/BS = bone formation rate per bone surface; MAR = mineral apposition rate.

To quantitate the bone formation, dynamic histomorphometric analysis was performed in 14‐week‐old mice after calcein double labeling spaced 5 days apart. A 23% decrease in MS/BS (*p* = 0.031; Fig. 5 *A*) and 34% decrease in BFR (*p* = 0.0007; Fig. 5 *B*) was observed in CRH‐Tg mice compared to WT mice. The MAR tended to be lower in CRH‐Tg mice relative to WT mice but was not statistically significant (*p* = 0.058; Fig. 5 *C*).

### Cortical bone strength is decreased in CRH‐Tg mice compared to WT mice

To determine whether the changes in cortical bone properties in CRH‐Tg mice affected bone strength, biomechanical properties were assessed by three‐point bending in the femur. The structural properties—ultimate load, energy to fail, and stiffness—were all reduced 38% to 41% in the CRH‐Tg mice compared to WT mice (Table 2). In contrast, the material properties—ultimate stress, toughness, and modulus—were not significantly reduced. These findings suggest that the decreased structural properties are not a result of differences in material properties, but could be due to the differences observed in the thickness of the cortical compartment in CRH‐Tg mice.

### CRH‐Tg mice have significant changes in bone turnover and bone formation genes

**Figure 6:**
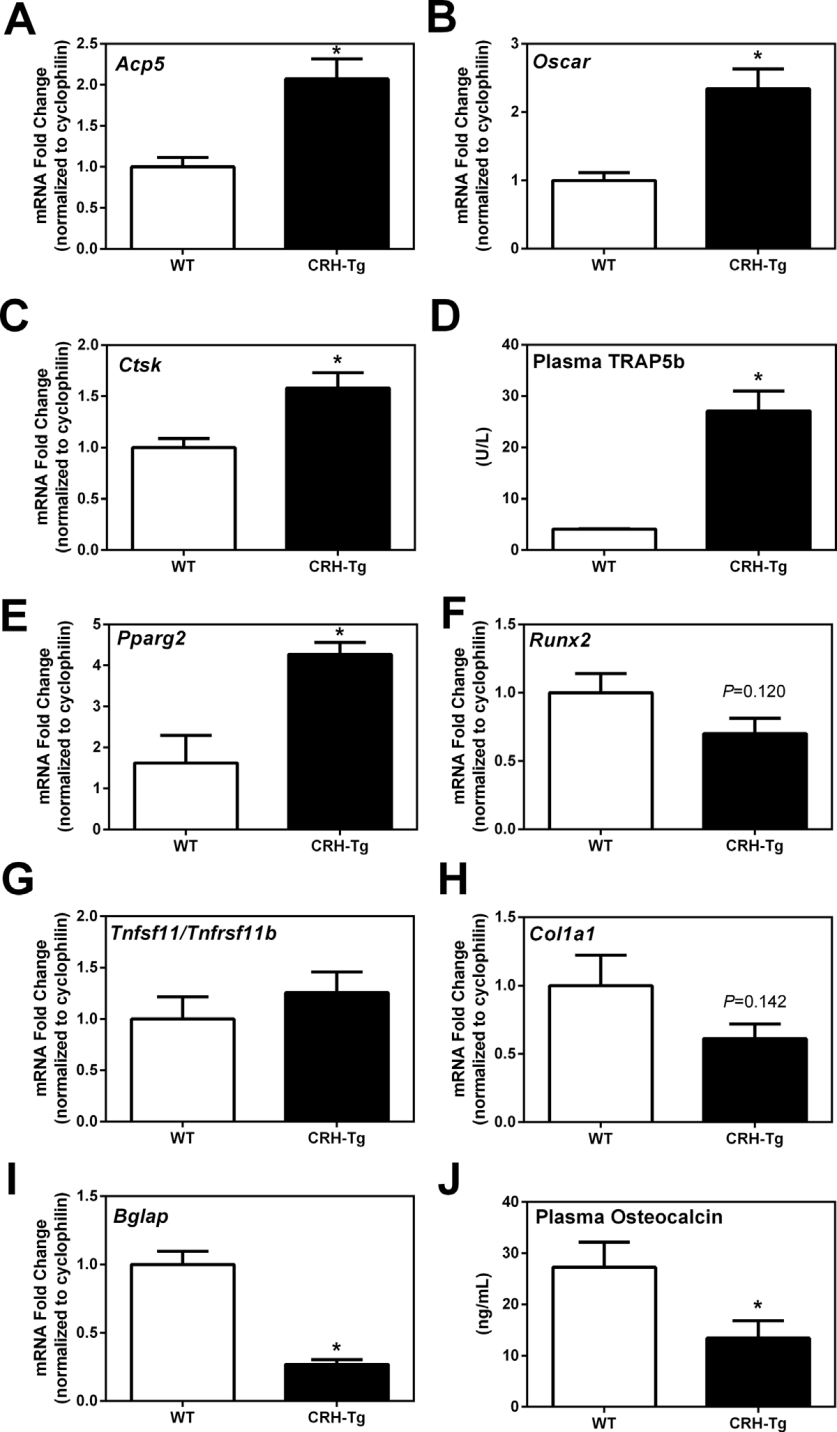
The expression of bone turnover genes is significantly increased and bone forming genes are significantly decreased in CRH‐Tg mice compared to WT mice. qPCR analysis of osteoclast markers: (*A*) TRAP (*Acp5*), (*B*) osteoclast associated receptor (*Oscar*), and (*C*) cathepsin K (*Ctsk*). (*D*) Plasma TRAP5b measured by ELISA. qPCR analysis of genes important in mesenchymal cell differentiation towards the adipocyte lineage (*E*) peroxisome proliferator‐activator receptor gamma‐2 (*PPARg2*), and (*F*) runt related transcription factor 2 (*Runx2*); (*G*) the ratio between receptor activator of nuclear factor kappa‐B ligand (*Tnfsf11*) and osteoprotegerin (*Tnfrsf11b*); and osteoblast markers: (*H*) collagen type 1 alpha 1 (*Col1a1*) and (*I*) osteocalcin (*Bglap*) from humeri of 13‐week‐old to 14‐week‐old male WT and CRH‐Tg mice. (*J*) Plasma osteocalcin measured by ELISA. All data shown have WT (*n* = 9) and CRH‐Tg (*n* = 6) except *D*, which has *n* = 5 per group. Data represent the average ± SE. *p < 0.05 WT versus CRH‐Tg using *t* test with Welch's correction. TRAP = tartrate resistant acid phosphatase.

To explore how changes observed may be mediated at the cellular level, gene expression was measured by qPCR in the humeri of WT and CRH‐Tg mice. The osteoclast associated markers TRAP (encoded by *Acp5*), *Oscar* (osteoclast associated receptor), and *Ctsk* (Cathepsin K) were significantly increased in CRH‐Tg mice compared to WT mice (*p *< 0.01; Fig. 6 *A*–*C*). In agreement with the gene expression data for *Acp5*, plasma TRAP5b levels were increased 6.5‐fold in CRH‐Tg mice compared to WT (*p* = 0.004; Fig. 6 *D*). These data suggest the CRH‐Tg mice have increased bone turnover relative to WT mice, a finding consistent with increased osteoclastogenesis by GCs.

To better understand the mechanism of increased adiposity of the bone in CRH‐Tg mice compared to WT mice, we measured the expression of two transcription factors that contribute to adipocyte versus osteoblast differentiation. *Pparγ2* is a transcription factor that shifts mesenchymal cell differentiation away from the osteoblast lineage and toward the adipocyte lineage. *Pparγ2* expression was increased fourfold in CRH‐Tg compared to WT mice (*p* < 0.0001; Fig. 6 *E*). The transcription factor *Runx2*, on the other hand, is responsible for driving mesenchymal cells toward the osteoblast lineage, and expression of this factor tended to be decreased (*p* = 0.120; Fig. 6 *F*). These data suggest that there is an increase in the transcription of factors that drive adipocyte differentiation, which complements the increase in adipocytes observed by histomorphometry.

Receptor activator of nuclear factor κ‐B ligand (RANKL, encoded by *Tnfsf11*) is a cytokine expressed by osteoblasts that acts on osteoclasts to increase their differentiation. Osteoprotegerin (OPG, encoded by *Tnfrsf11b*) is also secreted by osteoblasts and binds to RANKL, thus blocking RANKL signaling in osteoclasts. We measured the *Tnfsf11/Tnfrsf11b* ratio (RANKL/OPG mRNA ratio) and found no significant difference between WT and CRH‐Tg mice (Fig. 6 *G*). In contrast, the expression of osteoblast‐derived gene products important for bone formation was reduced 48% for *Col1a1* (*p* = 0.142; Fig. 6 *H*) and 77% for the gene encoding osteocalcin (*Bglap*; *p* < 0.0001; Fig. 6 *I*) in the CRH‐Tg mice compared to WT mice. At the protein level, plasma osteocalcin measured by ELISA was decreased 50% in the CRH‐Tg mice relative to WT mice (*p* = 0.0366; Fig. 6 *J*). Together, these findings are consistent with the decreased bone formation, as shown by our static and dynamic histomorphometric data.

### CRH‐Tg mice have increased expression of genes important for muscle breakdown

**Figure 7:**
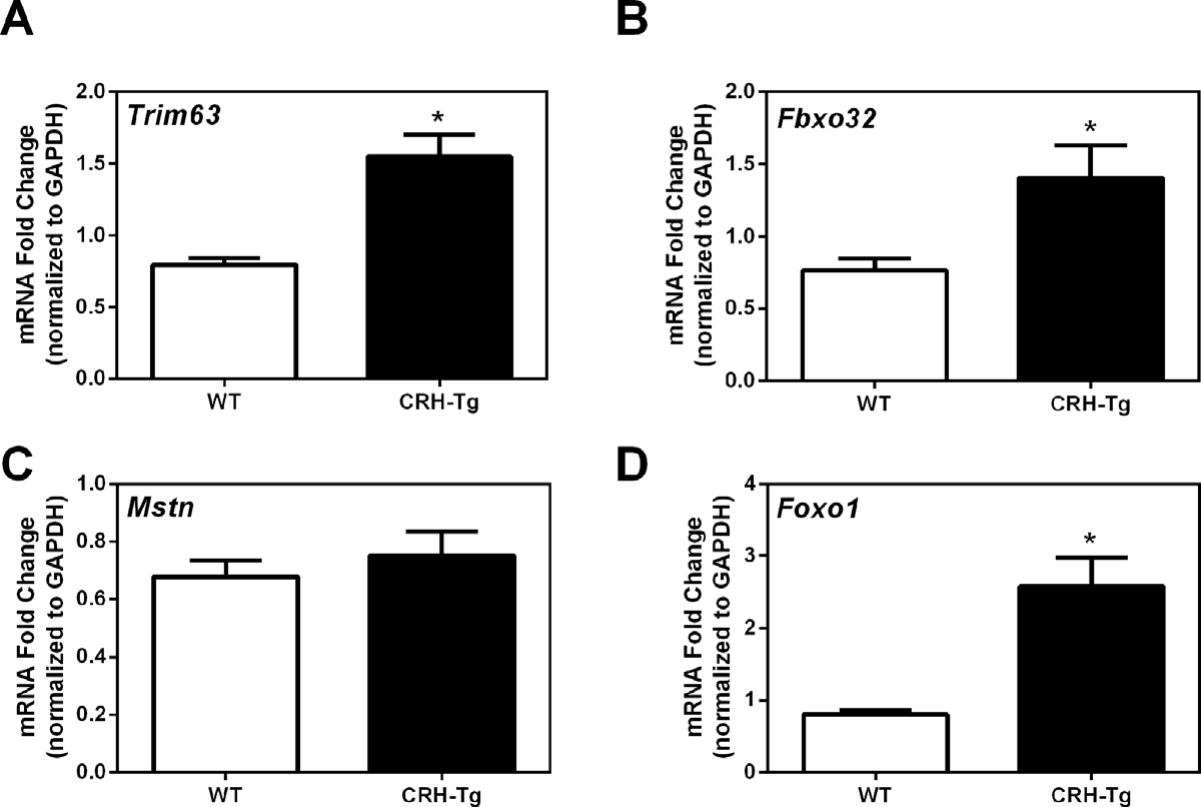
Muscle atrophy gene expression is significantly increased in CRH‐Tg mice compared to WT mice. qPCR analysis of genes important in increasing muscle breakdown: (*A*) muscle ring finger 1, Murf (*Trim63*), (B) muscle atrophy F‐box, Mafbx (*Fbxo32*), (*C*) myostatin (*Mstn*), and (*D*) forkhead box protein 1 (*Foxo1*) in gastrocnemius of 13‐week‐old to 14‐week‐old male WT (*n* = 8) and CRH‐Tg (*n* = 6) mice. Data represent the average ± SE. *p < 0.05 WT versus CRH‐Tg using *t* test with Welch's correction.

Muscle atrophy is another common side effect of chronic GC exposure, and muscle loss can contribute to increased fracture risk.^18^ To assess if muscle breakdown is increased in the CRH‐Tg mice relative to WT mice, we examined the expression of important muscle differentiation and atrophy genes. Two atrophy genes, *Trim63* (Murf‐1) and *Fbxo32* (Mafbx), were both increased 1.9‐fold in CRH‐Tg mice compared to WT mice (*p* < 0.05; Fig. 7 *A*, *B*). In contrast, there was no significant change in myostatin (*Mstn*), the negative growth differentiation factor (Fig. 7 *C*). MuRF1 and MAFbx are ubiquitin ligases that signal proteins for proteasomal degradation and are potently upregulated by glucocorticoids. Increased expression of these genes is associated with increased muscle breakdown.^19^ *Foxo1*, a transcription factor that binds to the upstream promoters of *Trim63* and *Fbxo32*, was increased fourfold in CRH‐Tg mice compared to WT mice (*p* = 0.0003; Fig. 7 *D*).

## Discussion

In the present study, we performed phenotypic characterization of the bones of CRH‐Tg mice and determined that they exhibit several properties consistent with GIO in humans with Cushing's syndrome. The CRH transgenic mouse, developed in 1992,^15^ has been previously characterized by other groups; however, these reports primarily focused on the metabolic, anti‐inflammatory, and behavioral effects of chronic corticosterone levels.^5^, ^20^, ^21^, ^22^, ^23^ A recent study by Harris and colleagues^16^ examined metabolic fluxes in CRH‐Tg mice, and reported reductions in CRH‐Tg total collagen synthesis in bone, whole‐body BMD, bone volume, and cortical thickness relative to WT mice, but did not report any trabecular bone parameters. Herein, we describe the detailed examination of the static and dynamic histomorphometric and molecular determinants of GIO, examining both cortical and trabecular bone to determine if this specific model of GC excess would lead to the range of anti‐osteogenic effects seen in Cushing's syndrome.

Cushing's syndrome patients present with both structural and functional impairments of the musculoskeletal system. Chronic GC exposure in Cushing's syndrome leads to a decreased function of osteoblasts, with a shift in the differentiation of bone marrow stromal cells away from osteoblasts and toward the adipocyte lineage in the bone.^24^, ^25^, ^26^ Consequently, Cushing's syndrome patients have decreased levels of circulating osteocalcin, an osteoblast‐derived factor.^27^ Concurrently, chronic GC exposure increases bone‐resorbing osteoclast survival.^28^, ^29^ Osteoblast and osteoclast markers are usually positively correlated because osteoblasts secrete important factors required for osteoclasts to function. However, in Cushing's syndrome and GIO, there is a documented uncoupling of this relationship.^30^ Mechanistically, this has been shown to be related to the direct effects of GCs on osteoblasts (to increase apoptosis and decrease differentiation to the osteoblast lineage^31^) and direct effects of GCs on osteoclasts (increasing osteoclast activity^32^, ^33^ and promoting osteoclast survival^33^, ^34^). The increase in osteoclasts by GCs is also thought to be due, in part, to the increased expression of the osteoclastogenic factor RANKL (*Tnfsf11*), and decreased expression of its decoy receptor OPG (*Tnfrsf11b*) by osteoblasts.^35^, ^36^, ^37^, ^38^, ^39^ Cushing's syndrome patients also develop muscle atrophy, which contributes to falls and fractures and may also contribute to weakening of the bone as a result of reduced bone loading stimulated by muscle contractions.^24^ The average mean bone densities in the forearm or proximal femur of Cushing's syndrome patients are 10% to 20% lower than controls.^40^, ^41^ In trabecular bone, BMD is decreased 16% to 40% in Cushing's syndrome,^40^, ^41^ a finding that is thought to account for the high incidence of vertebral and hip fractures in Cushing's patients.^27^, ^42^ However, there has also been a case report of appendicular skeletal fractures without vertebral fractures in Cushing's syndrome.^43^ Furthermore, when patients with similar BMD levels are compared, Cushing's patients are found to have a higher risk of fracture, suggesting that both BMD and bone quality need to be considered clinically.^24^

Baseline parameters in our CRH‐Tg mice derived from reconstituted embryos were similar to previous reports of the non‐cryopreserved animals. Despite having overall body weights that were similar, epididymal WAT weights were significantly increased in Cushing's mice relative to WT mice, consistent with what has been shown.^44^ Plasma corticosterone levels were approximately fivefold higher in the CRH‐Tg mice compared to WT mice. This increase was lower than the 10‐fold increase previously reported,^15^, ^45^ but this could be due to the difference in the time of blood collection between the studies (6 hours after start of light cycle versus 1 hour), because it is known that GC levels vary with circadian rhythm. As expected, the spleen weight in CRH‐Tg mice was significantly reduced compared to WT mice, highlighting the immunosuppressive effect of GCs.^46^ In contrast, the adrenal gland weights were significantly increased in CRH‐Tg mice, which is likely due to chronic ACTH activation because there is no negative feedback regulation of the HPA axis, and is consistent with what is observed in Cushing's disease caused by a pituitary adenoma.^47^

We next measured both the trabecular and cortical compartments by μCT in order to characterize the effects of chronic GC exposure on bone microarchitecture in this mouse model. vBMD in the cortical bone of the femur was significantly reduced in the CRH‐Tg mice. In contrast, vBMD of the trabecular compartment did not decrease despite a decrease in trabecular thickness and percent bone volume. This finding was interesting because, as aforementioned, GIO in humans generally results in more pronounced changes in the BMD of trabecular bone compared to cortical bone. However, in line with the CRH‐Tg model, several studies have shown significant decreases in cortical thickness with Cushing's syndrome^12^, ^14^, ^41^; and a very recent study using high‐resolution quantitative CT showed that patients with Cushing's syndrome exhibited deleterious effects primarily on cortical bone microstructure (lower cortical areas, thicknesses, and vBMD) when compared to age‐ and sex‐matched controls.^14^ The decreased trabecular thickness observed in CRH‐Tg mice is also in agreement with several studies that have shown exogenous administration of GC drugs causes trabecular thinning (reviewed in Dalle Carbonare and colleagues^48^).

The effect of exogenous GC drugs on the bones of mice have been investigated using different background strains and ages while varying the duration of GC treatment, resulting in disparate effects on trabecular versus cortical bone.^49^ For example, ICR mice treated with dexamethasone for 4 weeks show reduced trabecular bone volume with no change in cortical thickness,^50^ whereas C57BL/6 mice treated with dexamethasone for the same amount of time show increased trabecular bone and reduced cortical bone.^49^ Studies in which 5‐month‐old female C57Bl/6 mice were treated with prednisolone for 28 days, found that femoral cortical thickness^51^ and vertebral cortical and trabecular thickness were decreased relative to vehicle‐treated mice.^52^ A recent study found that when compared to CD‐1 mice, C57BL/6 mice are resistant to GIO.^53^ In addition, older mice are more prone to GIO relative to young mice.^54^ Although the CRH‐Tg mice are on a C57BL/6 background, we observed significant changes in both the trabecular and cortical compartments at only 14 weeks of age, suggesting that developmental and sustained exposure to GCs played an important role in the resulting phenotype.

We examined changes at the cellular level through static and dynamic histomorphometry. Adipocyte volume was significantly increased in the CRH‐Tg mice, which is expected with chronic GC exposure.^6^ Osteoblast numbers were not significantly decreased in CRH‐Tg mice compared to WT, whereas bone formation parameters (OV/BV, O.Wi., BFR/BS, and MS/BS) were decreased and osteoclast numbers were increased in the CRH‐Tg mice. From a mechanistic perspective, increases in osteoclast marker expression (*Acp5*, *Oscar*, and *Ctsk*) were consistent with the increased osteoclast numbers from the histomorphometry data. Although these markers suggest an increase in osteoclast activity, this idea would be strengthened by the measurement of a bone resorption marker downstream of the osteoclast, such as CTX. The decreased expression of osteoblast markers in CRH‐Tg mice, including *Bglap* and the osteoblastogenic transcription factor *Runx2*, did not correlate with the lack of significant change in osteoblast numbers by histomorphometry, suggesting that the osteoblasts which were present were less functional. The adipogenic transcription factor, *Pparγ2*, was increased and was consistent with the increased adipocytes in the bone marrow. Overall, these results highlight that chronic exposure to GCs in this model result in increased adipocytes and osteoclasts and a decrease in the normal function of osteoblasts in the bone.

Many reports of Cushing's syndrome highlight the dramatic decrease in osteocalcin (a bone formation marker) with conflicting reports of changes in bone resorption markers (reviewed in Toth and Grossman^55^). It should be noted that osteocalcin is not a perfect marker of osteoblast activity because it can also be released by osteoclastic resorption of the bone matrix and it is transcriptionally downregulated by GCs. Several publications do demonstrate significant increases in resorption markers with Cushing's syndrome,^27^, ^30^, ^41^, ^56^ consistent with the uncoupling phenomenon described above, whereas others either show no change or a slight decrease.^55^ It has been postulated that the discrepancies with respect to resorption markers and Cushing's disease could be because a specified GC threshold needs to be reached before resorption markers are detectable and this can vary with different subtypes of Cushing's syndrome.^55^ Despite controversy in the detection of changes in resorption markers, there is evidence from histomorphometric analysis of Cushing's patient samples that increased resorption is occurring (erosion surface was doubled^12^), consistent with what we observed in the CRH‐Tg model.

Mechanical testing of the femurs by three‐point bending revealed significant reductions in the structural properties of bone; however, the material properties were not significantly reduced. The decreased structural properties were likely due to reduced femur cortical thickness and not because of inherent material properties of the bone. These results suggest that CRH‐Tg mice would be more prone to fracture, and are consistent with the high fracture rates observed in Cushing's patients.^24^

Last, muscle atrophy markers were measured to evaluate the effects of chronic GC exposure on muscle, which can indirectly affect fracture risk. Because we found that muscle atrophy markers were increased in the CRH‐Tg mice, these results suggest that GC‐induced muscle atrophy can be induced at the gene expression level in this mouse model.

In conclusion, our results indicate that the CRH‐Tg mouse model has many features that are similar to that of Cushing's syndrome patients, including loss of trabecular bone volume, trabecular thinning, cortical thinning, and decreased osteoblast and increased osteoclast activity markers at the gene expression and cellular level. Together, these data indicate that 14‐week‐old CRH‐Tg mice are an appropriate model to study GIO and test new therapeutic interventions to mitigate bone loss.

## Disclosures

All authors state that they have no conflicts of interest.
